# Supplementary material for: Environmental flexibility does not explain metabolic robustness
Source: NPJ Syst Biol Appl. 2020 Nov 27;6:39. doi: 10.1038/s41540-020-00155-5 (PMC7695710; doi:10.1038/s41540-020-00155-5)
Supplement: Supplementary file 1 — Supplementary material [file 41540_2020_155_MOESM1_ESM.pdf]

Supplementary material to

Environmental flexibility does not explain metabolic robustness

Julian Libiseller-Egger,[1,2,*] Ben Coltman,[1,3] Matthias P. Gerstl,[1] and Jürgen Zanghellini[1,4,†]

[1] *Austrian Centre of Industrial Biotechnology, A-1190 Vienna, Austria*
[2] *University of Natural Resources and Life Sciences, A-1190 Vienna, Austria*
[3] *Department of Biotechnology, University of Natural Resources and Life Sciences, A-1190 Vienna, Austria*
[4] *Department of Analytical Chemistry, University of Vienna, A-1090 Vienna, Austria*

(Dated: October 2, 2020)

CONTENTS

List of Figures 1

List of Tables 1

Supplementary Notes 2
- Derivation of eq. (4) 2
- Derivation of eq. (5) 3
- Improving the approximation of eq. (6) 4
- Derivation of eq. (7) 5

Supplementary Figures 6

Supplementary Tables 14

References 18

LIST OF FIGURES

1 Speedup achieved by `pof2.0`. 6
2 Probability of failure (PoF) vs. carbon source molecular weight grouped by elemental composition. . . . 7
3 PoF and nutritional restriction. 8
4 PoF as function of the number of utilised carbon sources. 9
5 Impact of biomass composition on the PoF. 10
6 PoF of pathogens and non-pathogens. 11
7 Pairwise PoF differences for all *E. coli* pairs with equal $\boldsymbol{m_1}$ and $\boldsymbol{m_2}$ 12
8 Maximum error in the PoF estimate. 13

LIST OF TABLES

1 Evaluation of eq. (4) for the toy network in **Fig. 1a**. 14
2 List of loss-of-function (LOF) mutations that result in cell death for the toy network in **Fig. 1a**. 15
3 Minimal media. 15
4 Auxotrophic *E. coli* and *Shigella* strains and the respective supplements required for growth. 16
5 Auxotrophic *Salmonella* strains and the respective supplements required for growth. NMN, Nicotinamide mononucleotide 16
6 Fungal BioModels [1] accession IDs 17

SUPPLEMENTARY NOTES

Derivation of eq. (4)

In the following we expand on our previous work [2] and simplify eq. (2). Previously we showed that

$$f_d = \binom{r}{d}^{-1} \sum_{\emptyset \neq J \subseteq \{1,\ldots,m\}} (-1)^{|J|-1} \binom{r - |\mathscr{M}_{\mathcal{J}}|}{d - |\mathscr{M}_{\mathcal{J}}|}. \tag{1}$$

By inserting eqs. (1) and (3) into (2) and rearranging the sums, we obtain

$$F = \sum_{\emptyset \neq J \subseteq \{1,\ldots,m\}} (-1)^{|J|-1} \sum_{d=1}^{r} \binom{r - |\mathscr{M}_J|}{d - |\mathscr{M}_J|} p^d (1-p)^{r-d}. \tag{2}$$

For $d < |\mathscr{M}_J|$ the binomial coefficient is zero. Hence, the above equation becomes

$$F = \sum_{\emptyset \neq J \subseteq \{1,\ldots,m\}} (-1)^{|J|-1} \sum_{d=|\mathscr{M}_J|}^{r} \binom{r - |\mathscr{M}_J|}{d - |\mathscr{M}_J|} p^d (1-p)^{r-d}. \tag{3}$$

Finally, we introduce $i = d - |\mathscr{M}_J|$

$$F = \sum_{\emptyset \neq J \subseteq \{1,\ldots,m\}} (-1)^{|J|-1} p^{|\mathscr{M}_J|} \sum_{i=0}^{r-|\mathscr{M}_J|} \binom{r - |\mathscr{M}_J|}{i} p^i (1-p)^{r-|\mathscr{M}_J|-i} \tag{4}$$

and evaluate the sum over the full binomial distribution (which is equal to 1). Thus, we simply end up with eq. (4),

$$F = \sum_{\emptyset \neq J \subseteq \{1,\ldots,m\}} (-1)^{|J|-1} \; p^{|\mathscr{M}_J|}. \tag{5}$$

Derivation of eq. (5)

Suppose we know all m_1 essential reactions (minimal cut sets (MCSs) of cardinality 1) of a metabolic network. Then, eq. (4) becomes

$$\widetilde{F}^1 = \sum_{\emptyset \neq J \subseteq \{1,\ldots,m_1\}} (-1)^{|J|-1} \, p^{|\mathscr{M}_J|} \tag{6}$$

running over the power set of all m_1 MCSs. As those contain only one reaction each, the number of subsets with exactly i MCSs is given by the number of combinations $\binom{m_1}{i}$. Moreover, as no MCS of cardinality 1 has a common element with any other MCS, we can compute the cardinality of any union of MCSs by counting the number of elements. Thus, the sum above simplifies into

$$\widetilde{F}^1 = -\sum_{i=1}^{m_1} \binom{m_1}{i} (-p)^i = 1 - \sum_{i=0}^{m_1} \binom{m_1}{i} (-p)^i = 1 - (1-p)^{m_1}, \tag{7}$$

where we first complete the binomial expansion and then used the binomial identity. Note that this expression also considers the impact of all possible combinations of reaction deletions containing at least one essential reaction. As higher-cardinality MCSs only increase F further, eq. (7) represents a lower bound to the PoF.

Improving the approximation of eq. (6)

The PoF is given by

$$F = \sum_{d=1}^{r} w_d f_d \tag{8}$$

with

$$w_d = \binom{r}{d} p^d (1-p)^{r-d}. \tag{9}$$

Previously [2], we estimated F by truncating the sum over d from r to d_0. However, f_d increases monotonically with d, as any (higher-order) super-set that includes a MCS will also be lethal. Thus, any f_d for $d \geq d_0$ will be at least as large as f_{d_0} and eq. (8) can be approximated by (see **Supplementary Figure 8a**)

$$F \approx= \sum_{d=1}^{d_0} w_d f_d^{d_m} + f_{d_0}^{d_m} \sum_{d=d_0+1}^{r} w_d.$$

For very large d, $f_{d_0}^{d_m}$ might be smaller than the basal contributions of essential reactions, $f_{d_0}^{1}$. Thus by appropriately splitting the last sum and replacing f_{d_0} by f_d^1

$$F \approx \widetilde{F}_{d_0}^{d_m} = \sum_{d=1}^{d_0} w_d f_d^{d_m} + f_{d_0}^{d_m} \sum_{d=d_0+1}^{\rho} w_d + \sum_{d=\rho+1}^{r} w_d f_d^1, \tag{10}$$

the approximation of the PoF can be further improved. Here, the second sum is carried out over all $d_0 + 1 \leq d \leq \rho$ where $f_{d_0}^{d_m} \geq f_d^1$ is a more accurate estimator for the true f_d than f_d^1.

Derivation of eq. (7)

We assume that all MCS up to cardinality $d_m = 3$ have been computed, see **Supplementary Figure 8a**. Thus, all $f_d^{d_m}$ up do $d = 3$ are exact. At $d > 3$ we miss the contributions of higher-order MCSs. In the worst case all cut sets of cardinality four could already be lethal leading to $f_d^3 = 1$ for $d \geq 4$. The resulting maximum error is given by the weighted, cumulative sum of the hatched bars in **Supplementary Figure 8a** and can be computed by the compliment of the PoF estimate, $1 - \widetilde{F}_{d_0}^{d_m}$ minus the cumulated and weighted compliment of the error-free $f_d^{d_m}$s up to d_m,

$$\varepsilon_{\max} = 1 - \widetilde{F}_{d_0}^{d_m} - \sum_{d=0}^{d_m} w_d \left(1 - f_d^{d_m}\right). \tag{11}$$

In the worst case, i.e. if only essential reactions are known, eq. (11) simplifies into

$$\varepsilon_{\max}^1 = (1-p)^{m_1} - (1-p)^{r-1} \left[1 + p(r - m_1 - 1)\right]. \tag{12}$$

When added to eq. (7), the resulting expression gives an upper bound for F (**Supplementary Figure 8**). We note that the ratio

$$\frac{\varepsilon_{\max}^1}{1 - \widetilde{F}^1} = 1 - (1-p)^{r-m_1-1} \left[1 + p(r - m_1 - 1)\right], \tag{13}$$

which represents the error relative to the network's robustness, only depends on the mutation rate p and the number of non-essential reactions, $r - m_1$, see **Supplementary Figure 8c**.

SUPPLEMENTARY FIGURES

a **b**

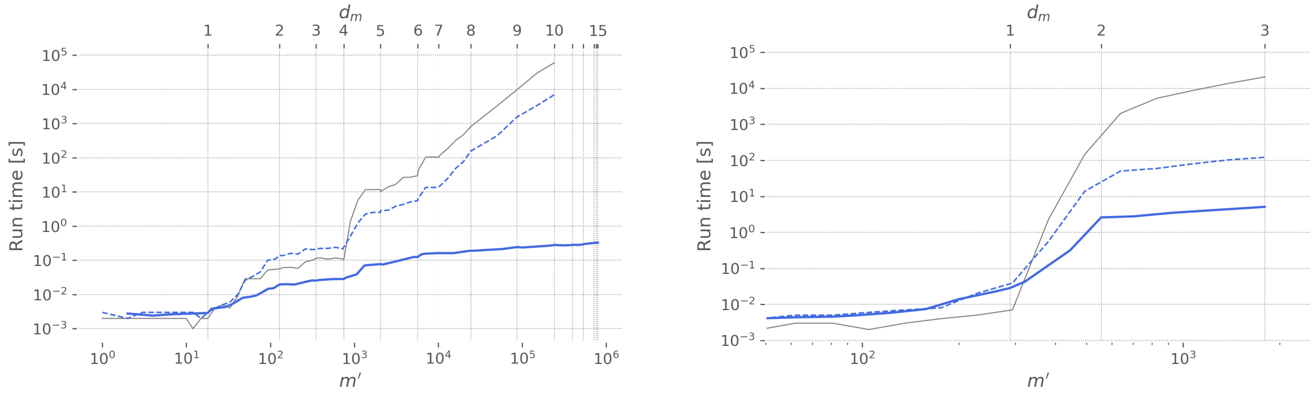

Supplementary Figure 1. **Speedup achieved by `pof2.0`.** Run time as function of the number of processed MCS, m_0, for `pof2.0` (blue) compared to the previous implementation (gray) [2]. We evaluated the total PoF for the CCMM of *E. coli* [3] with $d_m = 10$ and $d_0 = 15$ **a** as well as for the GSMM *i*JO1366 [4] with $d_m = 3$ and $d_0 = 8$ **b** simulating aerobic growth on glucose minimal medium. The blue solid and dashed lines illustrate performance of `pof2.0` for the linearly compressed and uncompressed networks, respectively.

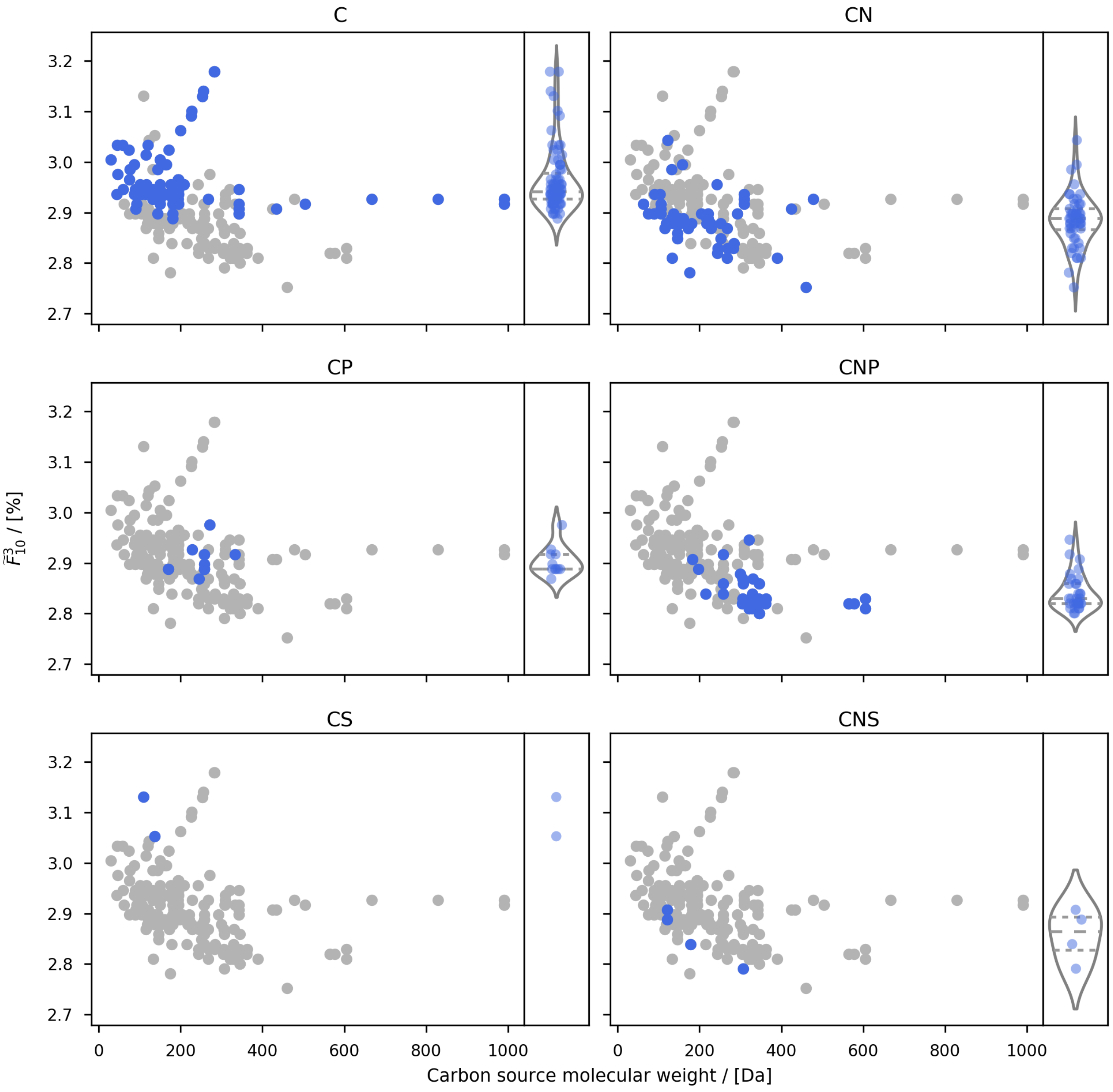

Supplementary Figure 2. **PoF vs. carbon source molecular weight grouped by elemental composition.** Each panel highlights the PoF for carbon sources exclusively containing elements as indicated by the panel title plus hydrogen and oxygen. Violin plots show the corresponding distributions.

a **b**

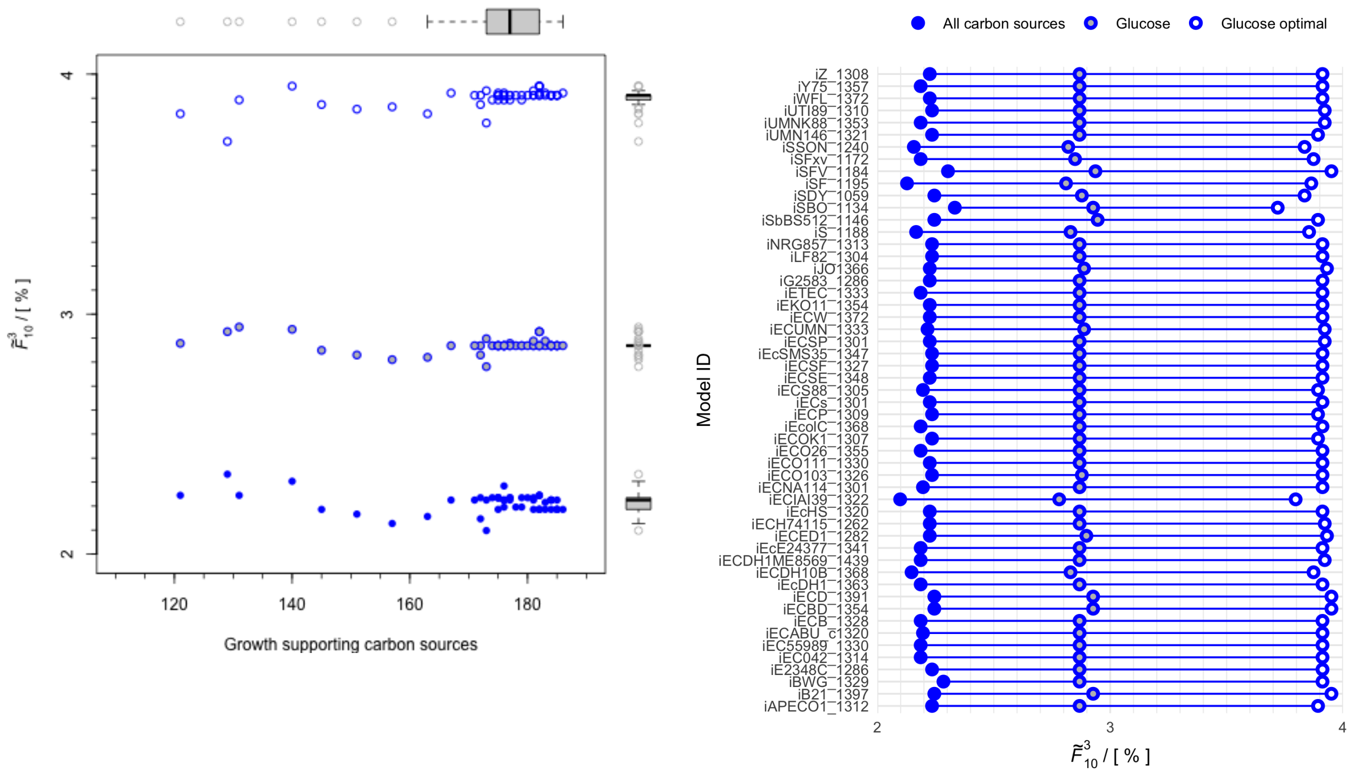

Supplementary Figure 3. **PoF and nutritional restriction.** **a**: PoF, as function of the number of growth-supporting environments for multiple GSMMs of *E. coli* and *Shigella* strains. Boxplots on top and to the right summarise the distribution of the data. **b**: PoF for optimal growth on glucose alone, any growth on glucose alone and any growth on all carbon sources present in the respective model. With the exception of *i*SBO_1134 variances among the models are fairly consistent across the three conditions.

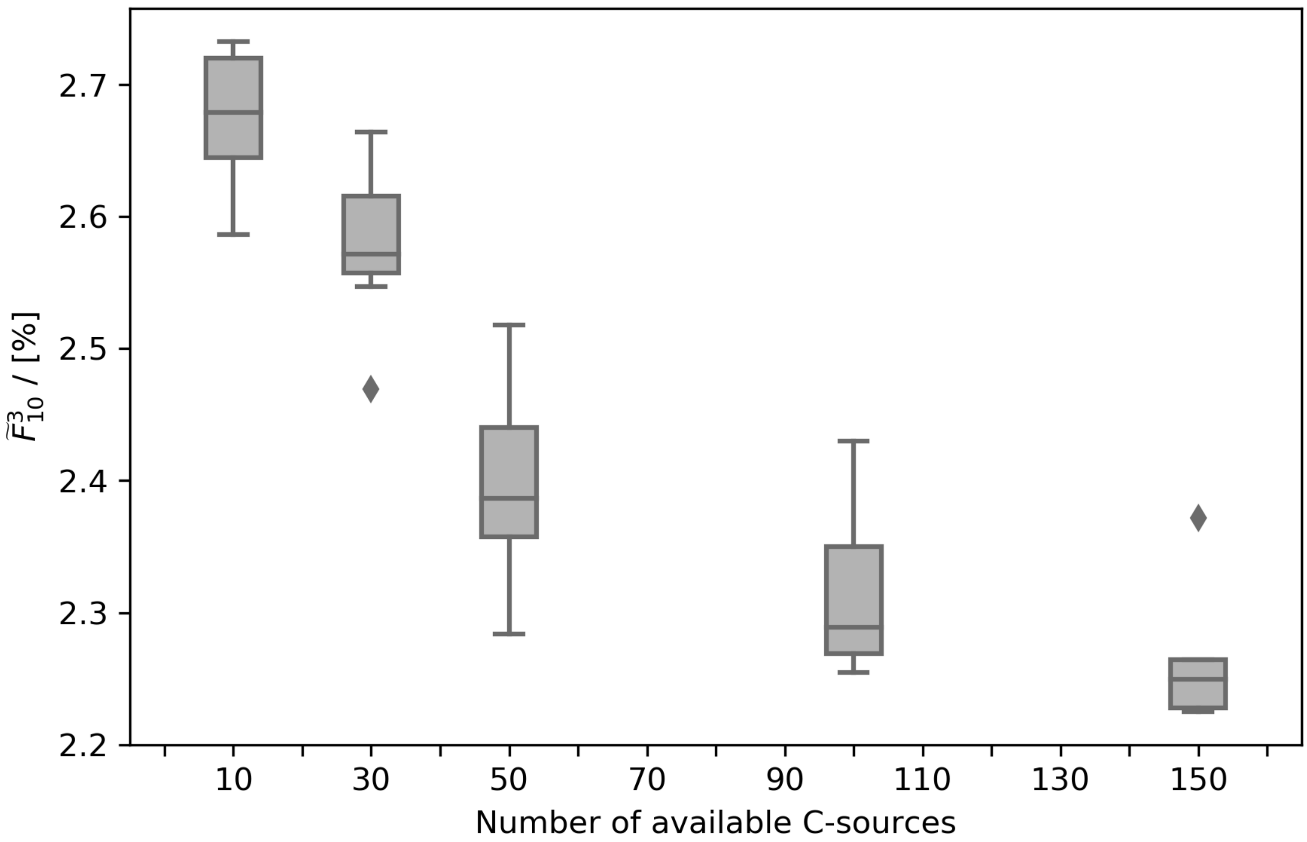

Supplementary Figure 4. **PoF as function of the number of utilised carbon sources.** Each box plot illustrates the distribution of the PoF in the GSMM *i*JO1366 across ten runs with randomly selected carbon sources.

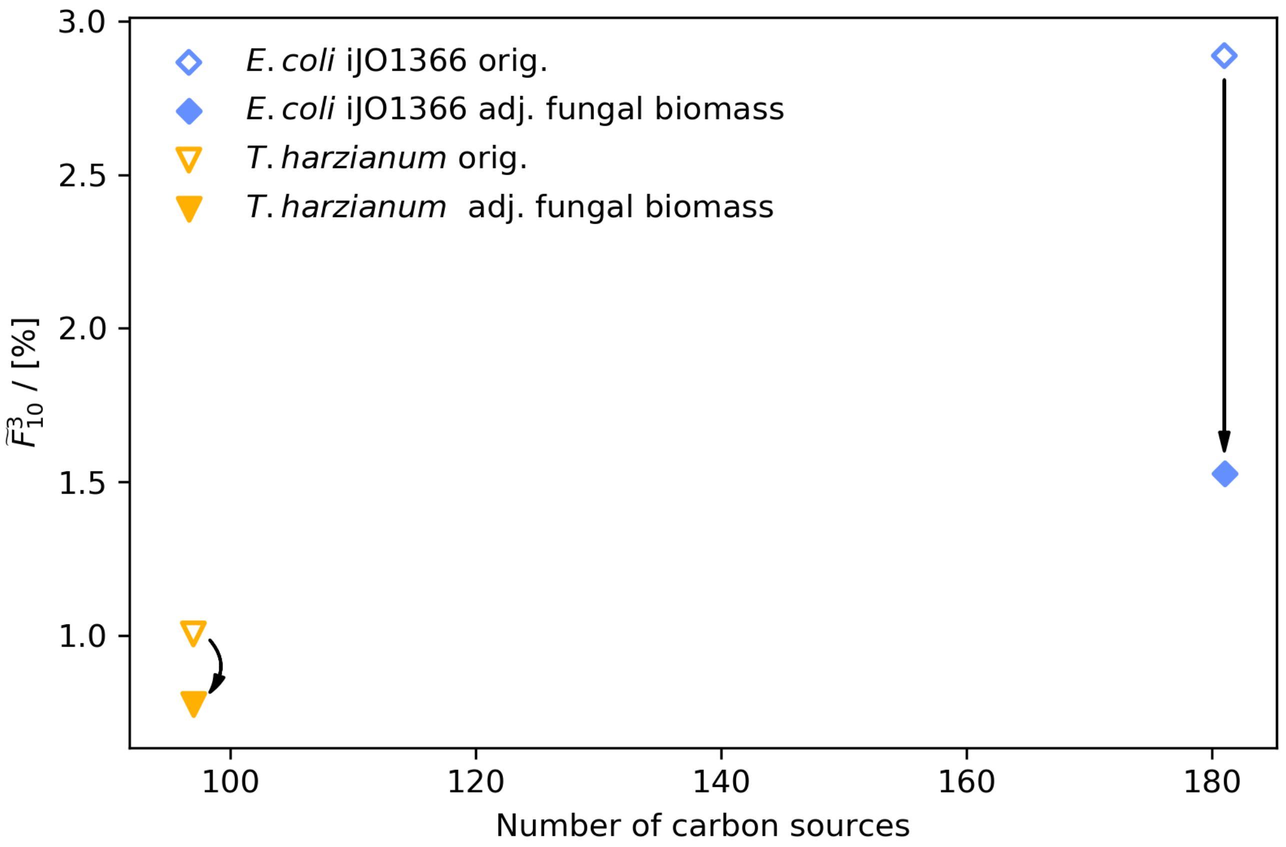

Supplementary Figure 5. **Impact of biomass composition on the PoF.** With its growth objective set to the (less detailed – 44 vs. 68 components) biomass reaction used by the fungal models, the *E. coli* GSMM *i*JO1366 showed a drastically decreased PoF. However, it should be noted that four fungal biomass components (ergosterol, zymosterol, chitin, and (1-3)-β-D-glucan) not present in *i*JO1366 had to be omitted. Removing these from the growth reaction for one of the fungal models (MODEL1604280000, *Trichoderma harzianum*) also reduced its PoF, but to a lesser extent.

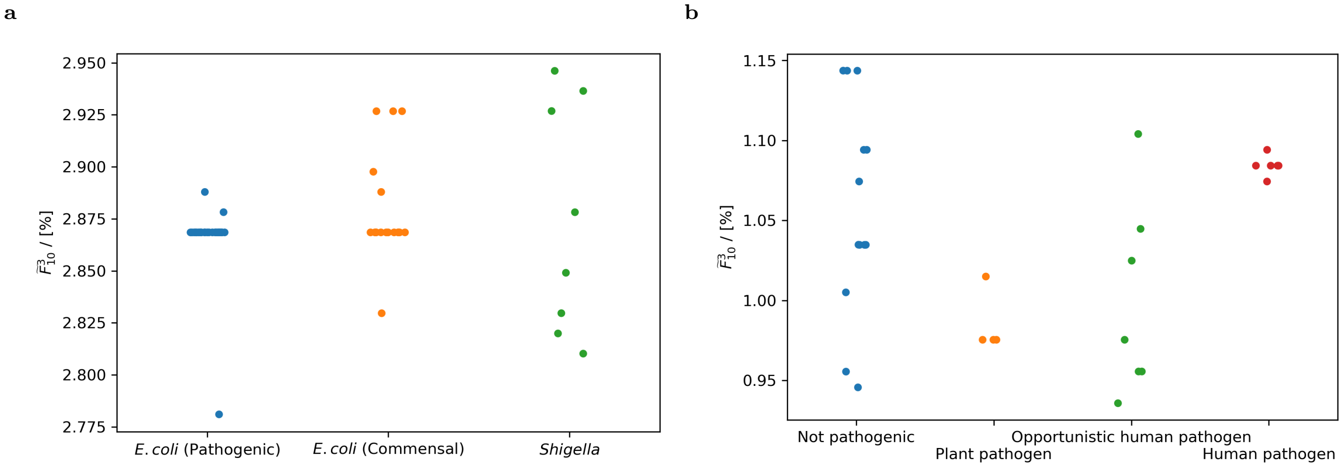

Supplementary Figure 6. **PoF of pathogens and non-pathogens.** *Shigella* and pathogenic *E. coli* strains do not exhibit different PoFs compared to non-pathogenic *E. coli* strains **a**. Overall, the fungal species show a similar picture with the variability of the non-pathogens essentially encompassing all other groups **b**. The groups of plant and human pathogens each cluster quite tightly. However, this does not allow to infer general trends given the small number of models and that both groups contained several closely related species (*Fusarium* for plant and *Candida* for human pathogens).

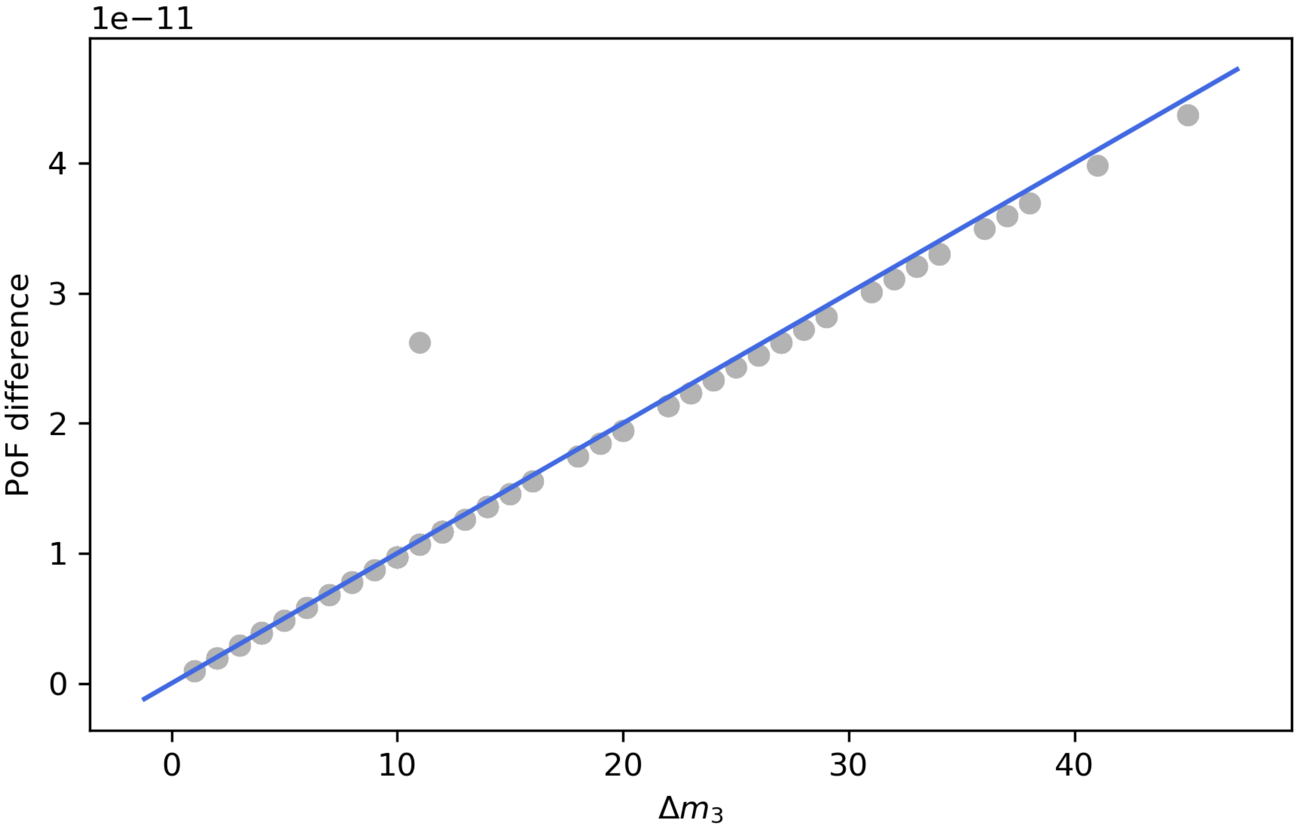

Supplementary Figure 7. **Pairwise PoF differences for all *E. coli* pairs with equal m_1 and m_2.** The blue line is given by eq. (5) with $p = 10^{-12}$.

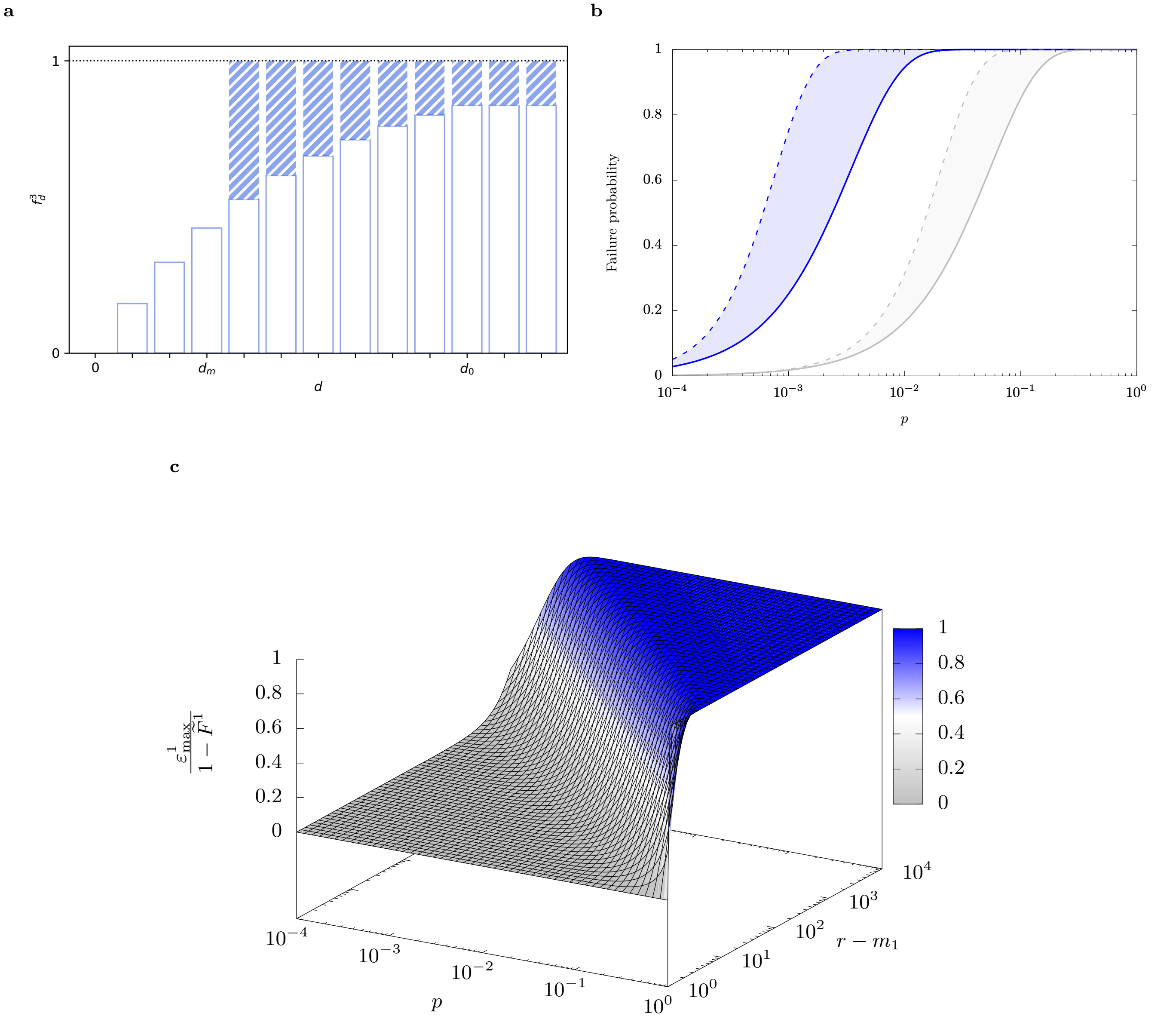

Supplementary Figure 8. **Maximum error in the PoF estimate.** **a** Visualisation of the maximum possible error (hatched bars) as function of d. Illustrated is a case with $d_m = 3$ and $d_0 = 10$. Note that for $d > d_m$, f_d^3 (empty bars) is only given by MCSs with cardinality up to 3. For $d > d_0$, we no longer update f_d^3, but use the value at $f_{d_0}^3$. **b** Lower ($\widetilde{F}^1$, solid lines) and upper ($\widetilde{F}^1 + \varepsilon^1_{\max}$, dashed lines) bounds of the PoF as function of the mutation rate p given the number of essential reactions m_1 for two *E. coli* models. The GSMM *i*JO1366 [4] with $r = 2583$ and $m_1 = 289$ is depicted in blue; a model of *E. coli*'s CCMM [3] ($r = 95$, $m_1 = 18$) in grey. The shaded area indicates maximal possible uncertainty in F. **c** $\varepsilon^1_{\max}/(1-\widetilde{F}^1)$ according to eq. (13) as function for p and $r - m_1$. The relative error grows with mutation rate and network size.

SUPPLEMENTARY TABLES

Supplementary Table 1. Evaluation of eq. (4) for the toy network in **Fig. 1a**.

i	MCS_i	$\mathcal{J}_i$	$\lvert\mathcal{J}_i\rvert$	$\mathscr{M}_{\mathcal{J}_i}$	$\lvert\mathscr{M}_{\mathcal{J}_i}\rvert$	$(-1)^{\lvert\mathcal{J}_i\rvert-1}p^{\lvert\mathscr{M}_{\mathcal{J}_i}\rvert}$	$\sum_{i'=1}^{i}(-1)^{\lvert\mathcal{J}_{i'}\rvert-1}p^{\lvert\mathscr{M}_{\mathcal{J}_{i'}}\rvert}$
1	$\{r_6\}$	$\{1\}$	1	$\{r_6\}$	1	$+p$	$+p$
2	$\{r_1, r_2\}$	$\{2\}$	1	$\{r_1, r_2\}$	2	$+p^2$	$+p + p^2$
3	$\{r_4, r_5\}$	$\{3\}$	1	$\{r_4, r_5\}$	2	$+p^2$	$+p + 2p^2$
4	$\{r_1, r_3, r_5\}$	$\{4\}$	1	$\{r_1, r_3, r_5\}$	3	$+p^3$	$+p + 2p^2 + p^3$
$m = 5$	$\{r_2, r_3, r_4\}$	$\{5\}$	1	$\{r_2, r_3.r_4\}$	3	$+p^3$	$+p + 2p^2 + 2p^3$
6		$\{1, 2\}$	2	$\{r_1, r_2, r_6\}$	3	$-p^3$	$+p + 2p^2 + p^3$
7		$\{1, 3\}$	2	$\{r_4, r_5, r_6\}$	3	$-p^3$	$+p + 2p^2$
8		$\{1, 4\}$	2	$\{r_1, r_3, r_5, r_6\}$	4	$-p^4$	$+p + 2p^2 - p^4$
9		$\{1, 5\}$	2	$\{r_2, r_3, r_4, r_6\}$	4	$-p^4$	$+p + 2p^2 - 2p^4$
10		$\{2, 3\}$	2	$\{r_1, r_2, r_4, r_5\}$	4	$-p^4$	$+p + 2p^2 - 3p^4$
11		$\{2, 4\}$	2	$\{r_1, r_2, r_3, r_5\}$	4	$-p^4$	$+p + 2p^2 - 4p^4$
12		$\{2, 5\}$	2	$\{r_1, r_2, r_3, r_4\}$	4	$-p^4$	$+p + 2p^2 - 5p^4$
13		$\{3, 4\}$	2	$\{r_1, r_3, r_4, r_5\}$	4	$-p^4$	$+p + 2p^2 - 6p^4$
14		$\{3, 5\}$	2	$\{r_2, r_3, r_4, r_5\}$	4	$-p^4$	$+p + 2p^2 - 7p^4$
15		$\{4, 5\}$	2	$\{r_1, r_2, r_3, r_4, r_5\}$	5	$-p^5$	$+p + 2p^2 - 7p^4 - p^5$
16		$\{1, 2, 3\}$	3	$\{r_1, r_2, r_4, r_5, r_6\}$	5	$+p^5$	$+p + 2p^2 - 7p^4$
17		$\{1, 2, 4\}$	3	$\{r_1, r_2, r_3, r_5, r_6\}$	5	$+p^5$	$+p + 2p^2 - 7p^4 + p^5$
18		$\{1, 2, 5\}$	3	$\{r_1, r_2, r_3, r_4, r_6\}$	5	$+p^5$	$+p + 2p^2 - 7p^4 + 2p^5$
19		$\{1, 3, 4\}$	3	$\{r_1, r_3, r_4, r_5, r_6\}$	5	$+p^5$	$+p + 2p^2 - 7p^4 + 3p^5$
20		$\{1, 3, 5\}$	3	$\{r_2, r_3, r_4, r_5, r_6\}$	5	$+p^5$	$+p + 2p^2 - 7p^4 + 4p^5$
21		$\{1, 4, 5\}$	3	$\{r_1, r_2, r_3, r_4, r_5, r_6\}$	6	$+p^6$	$+p + 2p^2 - 7p^4 + 4p^5 + p^6$
22		$\{2, 3, 4\}$	3	$\{r_1, r_2, r_3, r_4, r_5\}$	5	$+p^5$	$+p + 2p^2 - 7p^4 + 5p^5 + p^6$
23		$\{2, 3, 5\}$	3	$\{r_1, r_2, r_3, r_4, r_5\}$	5	$+p^5$	$+p + 2p^2 - 7p^4 + 6p^5 + p^6$
24		$\{2, 4, 5\}$	3	$\{r_1, r_2, r_3, r_4, r_5\}$	5	$+p^5$	$+p + 2p^2 - 7p^4 + 7p^5 + p^6$
25		$\{3, 4, 5\}$	3	$\{r_1, r_2, r_3, r_4, r_5\}$	5	$+p^5$	$+p + 2p^2 - 7p^4 + 8p^5 + p^6$
26		$\{1, 2, 3, 4\}$	4	$\{r_1, r_2, r_3, r_4, r_5, r_6\}$	6	$-p^6$	$+p + 2p^2 - 7p^4 + 8p^5$
27		$\{1, 2, 3, 5\}$	4	$\{r_1, r_2, r_3, r_4, r_5, r_6\}$	6	$-p^6$	$+p + 2p^2 - 7p^4 + 8p^5 - p^6$
28		$\{1, 2, 4, 5\}$	4	$\{r_1, r_2, r_3, r_4, r_5, r_6\}$	6	$-p^6$	$+p + 2p^2 - 7p^4 + 8p^5 - 2p^6$
29		$\{1, 3, 4, 5\}$	4	$\{r_1, r_2, r_3, r_4, r_5, r_6\}$	6	$-p^6$	$+p + 2p^2 - 7p^4 + 8p^5 - 3p^6$
30		$\{2, 3, 4, 5\}$	4	$\{r_1, r_2, r_3, r_4, r_5\}$	5	$-p^5$	$+p + 2p^2 - 7p^4 + 7p^5 - 3p^6$
$2^m - 1 = 31$		$\{1, 2, 3, 4, 5\}$	5	$\{r_1, r_2, r_3, r_4, r_5, r_6\}$	6	$+p^6$	$+p + 2p^2 - 7p^4 + 7p^5 - 2p^6 = F$

Supplementary Table 2. List of LOF mutations that result in cell death for the toy network in Fig. 1a. MCSs are highlighted in bold. Note that all elements are super-sets of at least one MCS. Coloured cells indicate the number of possible combinations of LOF mutations. For instance, at $d = 2$ seven out of 15 possible combinations disrupt growth. Finally, the lower part of the table evaluates the PoF, F, with the help of eqs. (2) and (3) and using $p = 0.1$.

i \ d	1	2	3	4	5	6
1	$\{\}$	$\{r_1, r_2\}$	$\{r_1, r_2, r_3\}$	$\{r_1, r_2, r_3, r_4\}$	$\{r_1, r_2, r_3, r_4, r_5\}$	$\{r_1, r_2, r_3, r_4, r_5, r_6\}$
2		$\{r_4, r_5\}$	$\{r_1, r_2, r_4\}$	$\{r_1, r_2, r_3, r_5\}$	$\{r_1, r_2, r_3, r_4, r_6\}$	
3		$\{r_1, r_6\}$	$\{r_1, r_2, r_5\}$	$\{r_1, r_2, r_3, r_6\}$	$\{r_1, r_2, r_3, r_5, r_6\}$	
4		$\{r_2, r_6\}$	$\{r_1, r_2, r_6\}$	$\{r_1, r_2, r_4, r_5\}$	$\{r_1, r_2, r_4, r_5, r_6\}$	
5		$\{r_3, r_6\}$	$\{r_1, r_3, r_5\}$	$\{r_1, r_2, r_4, r_6\}$	$\{r_1, r_3, r_4, r_5, r_6\}$	
6		$\{r_4, r_6\}$	$\{r_1, r_3, r_6\}$	$\{r_1, r_2, r_5, r_6\}$	$\{r_2, r_3, r_4, r_5, r_6\}$	
7		$\{r_5, r_6\}$	$\{r_1, r_4, r_6\}$	$\{r_1, r_3, r_4, r_5\}$		
8			$\{r_1, r_5, r_6\}$	$\{r_1, r_3, r_4, r_6\}$		
9			$\{r_2, r_3, r_4\}$	$\{r_1, r_3, r_5, r_6\}$		
10			$\{r_2, r_3, r_6\}$	$\{r_1, r_4, r_5, r_6\}$		
11			$\{r_2, r_4, r_6\}$	$\{r_2, r_3, r_4, r_5\}$		
12			$\{r_2, r_5, r_6\}$	$\{r_2, r_3, r_4, r_6\}$		
13			$\{r_3, r_4, r_6\}$	$\{r_2, r_3, r_5, r_6\}$		
14			$\{r_3, r_5, r_6\}$	$\{r_2, r_4, r_5, r_6\}$		
15			$\{r_4, r_5, r_1\}$	$\{r_3, r_4, r_5, r_6\}$		
16			$\{r_4, r_5, r_2\}$			
17			$\{r_4, r_5, r_3\}$			
18			$\{r_4, r_5, r_6\}$			
19						
20						
f_d	1/6	7/15	18/20	15/15	6/6	1/1
w_d	0.354294	0.098415	0.01458	0.001215	0.000054	0.000001
$\sum_{d'=1}^{d} w_{d'} f_{d'}$	0.059049	0.104976	0.11810	0.119313	0.119367	$0.119368 = F$

Supplementary Table 3. Minimal media.

Medium component	Reaction ID	*E. coli*, *Shigella*, *Salmonella*	fungi
Calcium	EX_ca2_e	+	-
Chloride	EX_cl_e	+	-
Co^{2+}	EX_cobalt2_e	+	-
Cu^{2+}	EX_cu2_e	+	-
Fe^{2+}	EX_fe2_e	+	-
D-Glucose	EX_glc__D_e	+	+
H_2O	EX_h2o_e	+	-
H^+	EX_h_e	+	-
K^+	EX_k_e	+	-
Mg	EX_mg2_e	+	-
Mn^{2+}	EX_mn2_e	+	-
Molybdate	EX_mobd_e	+	-
Ammonium	EX_nh4_e	+	+
Ni^{2+}	EX_ni2_e	+	-
O_2	EX_o2_e	+	+
Phosphate	EX_pi_e	+	+
Sulfate	EX_so4_e	+	+
Zinc	EX_zn2_e	+	-

Supplementary Table 4. Auxotrophic *E. coli* and *Shigella* strains and the respective supplements required for growth.

Strain	Model ID	Supplement	Exchange reaction ID
Shigella boydii Sb227	iSBO_1134	Thiamin, Niacin	EX_thm_e, EX_nac_e
Shigella flexneri 2002017	iSFxv_1172	Niacin	EX_nac_e
Shigella sonnei Ss046	iSSON_1240	Niacin	EX_nac_e
Shigella flexneri 5 str. 8401	iSFV_1184	Niacin	EX_nac_e
Shigella flexneri 2a str. 301	iSF_1195	Methionine, Niacin	EX_met__L_e, EX_nac_e
Escherichia coli UMN026	iECUMN_1333	Niacin	EX_nac_e
Shigella boydii CDC 3083-94	iSbBS512_1146	Thiamin	EX_thm_e
Shigella flexneri 2a str. 2457T	iS_1188	Niacin	EX_nac_e
Escherichia coli str. K-12 substr. DH10B	iECDH10B_1368	Leucin	EX_leu__L_e

Supplementary Table 5. Auxotrophic *Salmonella* strains and the respective supplements required for growth. NMN, Nicotinamide mononucleotide

Strain	Model ID	Supplement	Exchange reaction ID
EC20120219	YS__Enteritidis__EC20120219	Tryptophan	Auxotrophy_trp__L_e
ATCC_51960	YS__Albany__ATCC_51960	Xanthine	Auxotrophy_xan_e
EC20121750	YS__Enteritidis__EC20121750	Histidine	Auxotrophy_his__L_e
99_7863	YS__Paratyphi A__99_7863	NMN	Auxotrophy_nmn_e
EC20120597	YS__Enteritidis__EC20120597	Tryptophan	Auxotrophy_trp__L_e
SA20090877	YS__Enteritidis__SA20090877	Tryptophan	Auxotrophy_trp__L_e
EC20120229	YS__Enteritidis__EC20120229	Tryptophan	Auxotrophy_trp__L_e
EC20121753	YS__Enteritidis__EC20121753	Tryptophan	Auxotrophy_trp__L_e
[NA]	YS__Paratyphi A__9_65	NMN	Auxotrophy_nmn_e
01_1852	YS__Paratyphi A__01_1852	NMN	Auxotrophy_nmn_e
[NA]	YS__Paratyphi A__98_9652	NMN	Auxotrophy_nmn_e
SA20094521	YS__Enteritidis__SA20094521	Tryptophan	Auxotrophy_trp__L_e
EC20120686	YS__Enteritidis__EC20120686	Tryptophan	Auxotrophy_trp__L_e
EC20130348	YS__Enteritidis__EC20130348	Tryptophan	Auxotrophy_trp__L_e
U288	YS__Typhimurium__U288	Histidine	Auxotrophy_his__L_e
EC20121748	YS__Enteritidis__EC20121748	Tryptophan	Auxotrophy_trp__L_e
A103(ParaA)	YS__Paratyphi A__A103(ParaA)	NMN	Auxotrophy_nmn_e
SA19942384	YS__Enteritidis__SA19942384	Tryptophan	Auxotrophy_trp__L_e
EC20122031	YS__Enteritidis__EC20122031	Tryptophan	Auxotrophy_trp__L_e
SA20093421	YS__Enteritidis__SA20093421	Tryptophan	Auxotrophy_trp__L_e
SA20090435	YS__Enteritidis__SA20090435	Tryptophan	Auxotrophy_trp__L_e
A61_149	YS__Paratyphi A__A61_149	NMN	Auxotrophy_nmn_e
EC20090195	YS__Enteritidis__EC20090195	Tryptophan	Auxotrophy_trp__L_e
SA19930684	YS__Enteritidis__SA19930684	Tryptophan	Auxotrophy_trp__L_e
138_69	YS__Paratyphi A__138_69	NMN	Auxotrophy_nmn_e

Supplementary Table 6. Fungal BioModels [1] accession IDs

Accession ID	Species
MODEL1604280000	Trichoderma harzianum
MODEL1604280001	Penicillium chrysogenum
MODEL1604280003	Fusarium verticillioides
MODEL1604280004	Trichoderma citrinoviride
MODEL1604280005	Chaetomium globosum
MODEL1604280006	Candida tropicalis
MODEL1604280007	Pichia guilliermondii
MODEL1604280010	Trichoderma longibrachiatum
MODEL1604280012	Aspergillus oryzae
MODEL1604280013	Magnaporthe grisea
MODEL1604280016	Aspergillus clavatus
MODEL1604280017	Yarrowia lipolytica
MODEL1604280018	Fusarium oxysporum
MODEL1604280019	Aspergillus terreus
MODEL1604280022	Trichoderma asperellum
MODEL1604280024	Trichoderma reesei
MODEL1604280025	Nectria haematococca
MODEL1604280026	Schizosaccharomyces japonicus
MODEL1604280028	Debaryomyces hansenii
MODEL1604280029	Aspergillus fumigatus
MODEL1604280031	Fusarium graminearum
MODEL1604280033	Candida glabrata
MODEL1604280038	Trichoderma virens
MODEL1604280039	Lodderomyces elongisporus
MODEL1604280041	Schizosaccharomyces pombe
MODEL1604280043	Candida lusitaniae
MODEL1604280046	Neurospora crassa
MODEL1604280049	Pichia stipitis
MODEL1604280052	Candida albicans
MODEL1604280055	Pichia pastoris

* julian.libiseller-egger@boku.ac.at
† juergen.zanghellini@univie.ac.at

[1] R. S. Malik-Sheriff, M. Glont, T. V. N. Nguyen, K. Tiwari, M. G. Roberts, A. Xavier, M. T. Vu, J. Men, M. Maire, S. Kananathan, E. L. Fairbanks, J. P. Meyer, C. Arankalle, T. M. Varusai, V. Knight-Schrijver, L. Li, C. Dueñas-Roca, G. Dass, S. M. Keating, Y. M. Park, N. Buso, N. Rodriguez, M. Hucka, and H. Hermjakob, Nucleic Acids Research 10.1093/nar/gkz1055 (2019).
[2] M. P. Gerstl, S. Klamt, C. Jungreuthmayer, and J. Zanghellini, Bioinformatics **32**, 730 (2016).
[3] J. D. Orth, R. M. T. Fleming, and B. O. Palsson, EcoSal Plus **4**, 10.1128/ecosalplus.10.2.1 (2010).
[4] J. D. Orth, T. M. Conrad, J. Na, J. A. Lerman, H. Nam, A. M. Feist, and B. Ø. Palsson, Mol Syst Biol **7**, 535 (2011).
